# SP-YOLO: A Real-Time and Efficient Multi-Scale Model for Pest Detection in Sugar Beet Fields

**DOI:** 10.3390/insects16010102

**Published:** 2025-01-20

**Authors:** Ke Tang, Yurong Qian, Hualong Dong, Yuning Huang, Yi Lu, Palidan Tuerxun, Qin Li

**Affiliations:** 1 School of Software, Xinjiang University, Urumqi 830091, China; 107552301689@stu.xju.edu.cn (K.T.); 107552301674@stu.xju.edu.cn (H.D.); 107552204759@stu.xju.edu.cn (Y.H.); 2 Key Laboratory of Signal Detection and Processing in Xinjiang Uygur Autonomous Region, Xinjiang University, Urumqi 830046, China; 3 Key Laboratory of Software Engineering, Xinjiang University, Urumqi 830091, China; 4 School of Computer Science and Technology, Xinjiang University, Urumqi 830046, China; outman@stu.xju.edu.cn; 5 School of Computer Science and Technology, Xinjiang Normal University, Urumqi 830054, China; pldtrs@xjnu.edu.cn; 6 College of Life Science and Technology, Xinjiang University, Urumqi 830017, China; liqptero@163.com

**Keywords:** sugar beet pest, pest detection, multi-scale feature fusion, deep learning, intelligent pest management

## Abstract

Beet crops are highly vulnerable to pest infestations throughout their growth cycle, which significantly affects crop development and yield. Timely and accurate pest identification is crucial for implementing effective control measures. Current pest detection tasks face two primary challenges: first, pests frequently blend into their environment due to similar colors, making it difficult to capture distinguishing features in the field; second, pest images exhibit scale variations under different viewing angles, lighting conditions, and distances, which complicates the detection process. This study constructed the BeetPest dataset, a multi-scale pest dataset for beets in complex backgrounds, and proposed the SP-YOLO model, which is an improved real-time detection model based on YOLO11. The model integrates a CNN and transformer (CAT) into the backbone network to capture global features. The lightweight depthwise separable convolution block (DSCB) module is designed to extract multi-scale features and enlarge the receptive field. The neck utilizes the cross-layer path aggregation network (CLPAN) module, further merging low-level and high-level features. SP-YOLO effectively differentiates between the background and target, excelling in handling scale variations in pest images. In comparison with the original YOLO11 model, SP-YOLO shows a 4.9% improvement in mean average precision (mAP@50), a 9.9% increase in precision, and a 1.3% rise in average recall. Furthermore, SP-YOLO achieves a detection speed of 136 frames per second (FPS), meeting real-time pest detection requirements. The model demonstrates remarkable robustness on other pest datasets while maintaining a manageable parameter size and computational complexity suitable for edge devices.

## Simple Summary

Sugar beet is a crucial raw material for sugar production, but pest infestations severely impact both its yield and quality. Therefore, rapid and accurate pest detection is essential for ensuring its production. Traditional manual detection methods heavily rely on the expertise of specialists, making them time-consuming and labor-intensive. With the advancement of computer vision technology, deep convolutional neural networks have been widely applied in pest detection. This paper presents a multi-scale, real-time pest detection model designed to enhance detection accuracy while reducing the model’s parameter size and computational load. Experimental results demonstrate that the proposed method outperforms existing approaches on the beet pest dataset, achieving higher detection accuracy and faster processing speed. This approach provides an effective technical solution and theoretical foundation for intelligent pest management.

## 1. Introduction

Sugar beet is the primary source of sucrose in temperate climates [1]. Throughout its growth cycle, sugar beet is susceptible to various insect pests, which can significantly reduce crop yield. Beetworms feed on above-ground plant material in their adult stage, while in the larval (or “wakame”) stage, they primarily target root sap below ground [2]. In addition to causing direct damage to the plant by feeding on both the leaves and roots, beetworm pests also serve as vectors for the transmission of pathogens [2,3]. Therefore, effective pest control is essential for optimizing sugar beet production.

Traditional pest detection methods typically rely on identification based on the experience of entomologists or technicians. These approaches are highly dependent on subjective experience, labor-intensive, and not scalable for large-scale applications [4,5]. Computer vision technology, however, offers a promising solution for the automated detection of insect pests. In recent years, pest identification techniques based on traditional machine learning have made significant progress, such as support vector machine (SVM), adaptive boosting (AdaBoost), artificial neural networks (ANNs), decision trees, and multiple linear regression (MLR) [6]. For example, ref. [7] proposed the machine vision system LOSS V2 algorithm for the initial identification of pests, followed by a scale-invariant feature transform (SIFT) for feature extraction [8]. Ref. [9] leveraged correlation-based feature selection combined with artificial neural networks to accurately classify eight tea pests. Other studies have also successfully applied improved SVM algorithms for classifying various vegetable pests [5,10]. Despite these advancements, traditional machine learning methods often require manual feature design, which makes it challenging to extract effective features. This can lead to issues such as inadequate model fitting, low robustness, and poor generalization performance [4].

**Figure 1:**
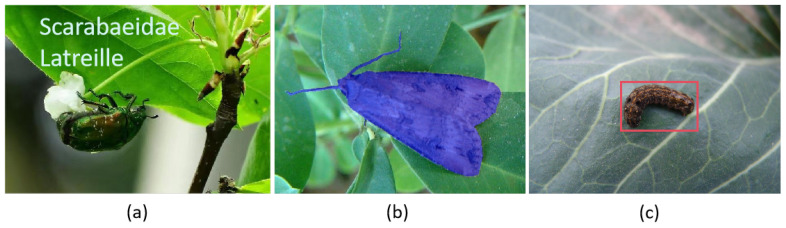
Comparison of three methods for insect pest identification based on convolutional neural networks: (**a**) classification, (**b**) segmentation, (**c**) object detection. The red box in the figure represents the pest detected.

With the development of deep learning, the convolutional neural network (CNN) has demonstrated outstanding performance in feature extraction, enabling automatic learning of pest characteristics without the need for human intervention. Currently, there are three main approaches for pest detection using deep convolutional neural networks: classification, segmentation, and object detection, as illustrated in Figure 1. The classification method is fast but is limited by its inability to handle multiple pest instances in a single image. The segmentation method divides the pest image into several regions with different properties, allowing for the separation of the pest from the background, with pest boundaries defined at the pixel level. This approach offers finer granularity, but it is cumbersome to manually create segmentation datasets, which can impact model accuracy and slow down inference speed [11].

Unlike classification and segmentation methods, the objective of object detection algorithms is not only to identify the object classes within an image but also to determine their positions and extents. Two-stage models include R-CNN [12], Fast R-CNN [13], and Faster R-CNN [14]. One-stage object detection algorithms perform end-to-end processing without the intermediate step of candidate region generation. Notable single-stage models include SSD [15], RetinaNet [16], Fcos [17], and the YOLO series. Two-stage algorithms tend to have slower inference speeds due to the generation of numerous candidate regions; however, their accuracy is typically higher as a result of this more detailed process. Single-stage algorithms, while faster, generally offer lower accuracy compared to their two-stage counterparts [18].

Object detection algorithms are increasingly employed in the field of agricultural pest detection. Object detection algorithms are increasingly utilized in the field of agricultural pest detection due to their broad applicability. For instance, ref. [19] employed Faster R-CNN, SSD, YOLOv3, and EfficientNet models for detecting dense aphid populations to facilitate pest control. Ref. [20] used YOLOv4 for early warning of pests in grain storage facilities, reducing the economic losses associated with food storage. Ref. [4] improved Faster R-CNN to detect tiny pests in greenhouse agriculture. Ref. [21] enhanced the YOLO model to detect pests in hazelnut orchards, enabling the development of a data-driven pest detection system. These models can efficiently identify targets and locate their positions and boundaries in images, addressing the key challenges of pest identification and localization in agricultural monitoring. The selection of models is based on the need to balance detection speed, accuracy, and the limitations of edge devices in different scenarios.

However, there are three key challenges associated with implementing pest detection models in complex real-world scenarios, as summarized in Table 1. First, when deploying models on edge devices, the large number of model parameters, high computational demands, and slow inference speeds make such models unsuitable for practical field applications. Second, many pest detection models are trained on a limited variety of pest species and at a single scale, resulting in poor generalization to diverse pest types or variable field conditions. Third, the simplicity of the training dataset background often leads to overfitting, where the model learns only to recognize basic environmental features, impairing its ability to adapt to the dynamic and complex conditions encountered in field environments.

Specifically, ref. [22] designed a hybrid counting network architecture to count sugar beet aphids. However, this approach overlooks the issue of model parameter redundancy, which hinders its deployment on mobile devices. Ref. [27] proposed a lightweight model based on YOLOv5 that demonstrated strong accuracy in detecting cotton insect pests. Nevertheless, the model was trained on data from only three cotton pest species, and its performance in recognizing other pest species remains unvalidated. To enhance the detection of shallow features in the original FPNet framework, ref. [11] incorporated a channel attention module into YOLOv5s. However, the evaluation of this approach was limited to the RTB dataset [32], which was collected under controlled laboratory conditions with a narrow range of pest species and a small-scale setup. Consequently, the model’s generalizability to real-world agricultural scenarios—characterized by a wider variety of pest species and variable environmental conditions—remains uncertain. Thus, further validation in more diverse and representative agricultural contexts is essential to evaluate the robustness and applicability of the proposed method in practical situations.

In this study, a novel lightweight and high-accuracy model for the rapid detection of sugar beet pests in complex field environments is proposed. The aforementioned challenges are addressed by constructing a comprehensive dataset for sugar beet pest detection, which includes multiple categories, varying scales, and complex background information.

The main contributions of this study are as follows:

(1) A new sugar beet dataset is presented, containing 12 common pest species and 1 type of pest trace, covering a range of scenes and scales.
(2) A lightweight pest detection model, SP-YOLO, is introduced. This model incorporates a new backbone network combining a CNN and transformer, which improves its ability to capture long-range dependencies. Additionally, a DSCB module is designed for various pest sizes, improving detection accuracy, and the path aggregation network is further improved for multi-scale feature fusion. Extensive experimental results demonstrate that the optimized SP-YOLO model outperforms existing object detection models in terms of accuracy while maintaining a smaller number of parameters and lower computational cost, enabling real-time object detection.

## 2. Materials

### 2.1. Image Data Acquisition

This study uses sugar beet pest images as a case study for model evaluation. Due to the lack of publicly available datasets for sugar beet pests, we created a custom dataset, named BeetPest, as described in Table 2. The dataset construction prioritized three key aspects: the diversity of pest species, variation in pest scales, and complex and variable backgrounds. Existing datasets typically fail to meet all three criteria simultaneously. Consequently, the BeetPest dataset was constructed from three sources: captured from real field conditions, retrieved via a search engine, and the publicly available IP102 dataset [33].

**Figure 2:**
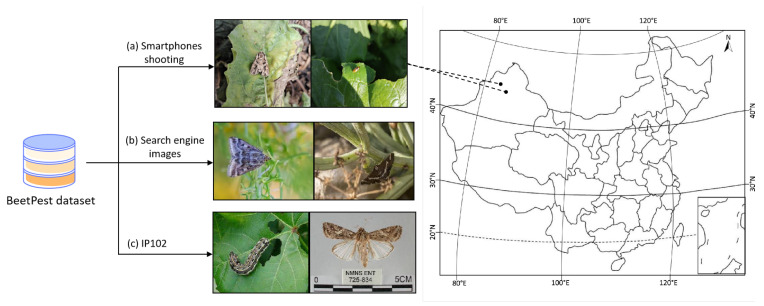
BeetPest dataset with three data sources: IP102 (specific class selection), internet images, and field photographs captured with smartphones. Pest infestation locations visualized on the map.

The image dataset includes 12 common beet pests and 1 type of pest trace, *Pegomyia hyosciami* (Panzer, 1809), totaling 5318 beet pest images, as shown in Figure 2. In collaboration with experts from the College of Life Science and Technology, Xinjiang University, we captured 765 images in beet fields and from pest specimens between April and August 2024. The images were taken using smartphones at two locations: Location 1, Emin County, Tacheng Region (Latitude: 46.81, Longitude: 83.85), and Location 2, Changji City, Changji Hui Autonomous Prefecture (Latitude: 44.00, Longitude: 87.02). The resolution of the images exceeded 2000 × 2000 pixels. Smartphone shooting images deliberately capture the scale variation in pest infestations and the complexity of background diversity in agricultural environments.

Due to the limited number of pest images captured in the field, primarily because farmers indiscriminately apply pesticides using UAV drones, additional images were sourced from the internet. To ensure dataset quality, we collected images from popular search engines—Google, Bing, Baidu, and Naver—which include 11 categories and a total of 2273 images. Additionally, we complemented these with pest images from the publicly available IP102 dataset. The IP102 dataset contains over 17,000 images across 102 pest categories. However, it was found to have significant labeling issues and a severe class imbalance. As a result, we selected 7 categories from the IP102 dataset, consisting of 2280 images. Each image was manually reviewed to confirm correct labeling, ensuring that mislabeled or redundant images were excluded from the final dataset.

**Figure 3:**
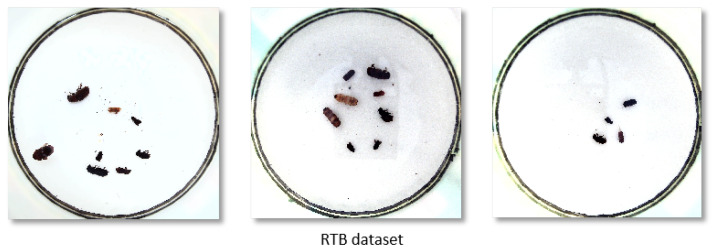
Samples of the RTB dataset, captured in a controlled experimental environment, including six invasive pests of pine trees.

To verify the robustness and generality of the model, we evaluated SP-YOLO on the RTB dataset [32], which comprises a total of 2183 images of six common invasive pests of pine trees, as illustrated in Figure 3. The dataset was generated by immersing the pests in alcohol in a controlled laboratory environment and capturing images of their various morphological characteristics. The pests in this dataset exhibit significant morphological similarities, which presents a challenging scenario for pest detection.

### 2.2. Creation of Dataset

The object detection task requires accurate image labeling, which can be challenging due to the large number of pest species and their varying morphologies. To minimize labeling errors, we collaborated with an expert in sugar beet pest identification to create the dataset. Labeling was performed using LabelImg, a tool available at https://github.com/HumanSignal/labelImg (accessed on 1 August 2024), and the dataset was formatted in the COCO format. Each label includes the pest species, the coordinates of the instance’s center, and the width and height of the bounding box. The dataset was then split into training, validation, and test sets in an 8:1:1 ratio.

## 3. Method

### 3.1. Baseline Model YOLO11

**Figure 4:**
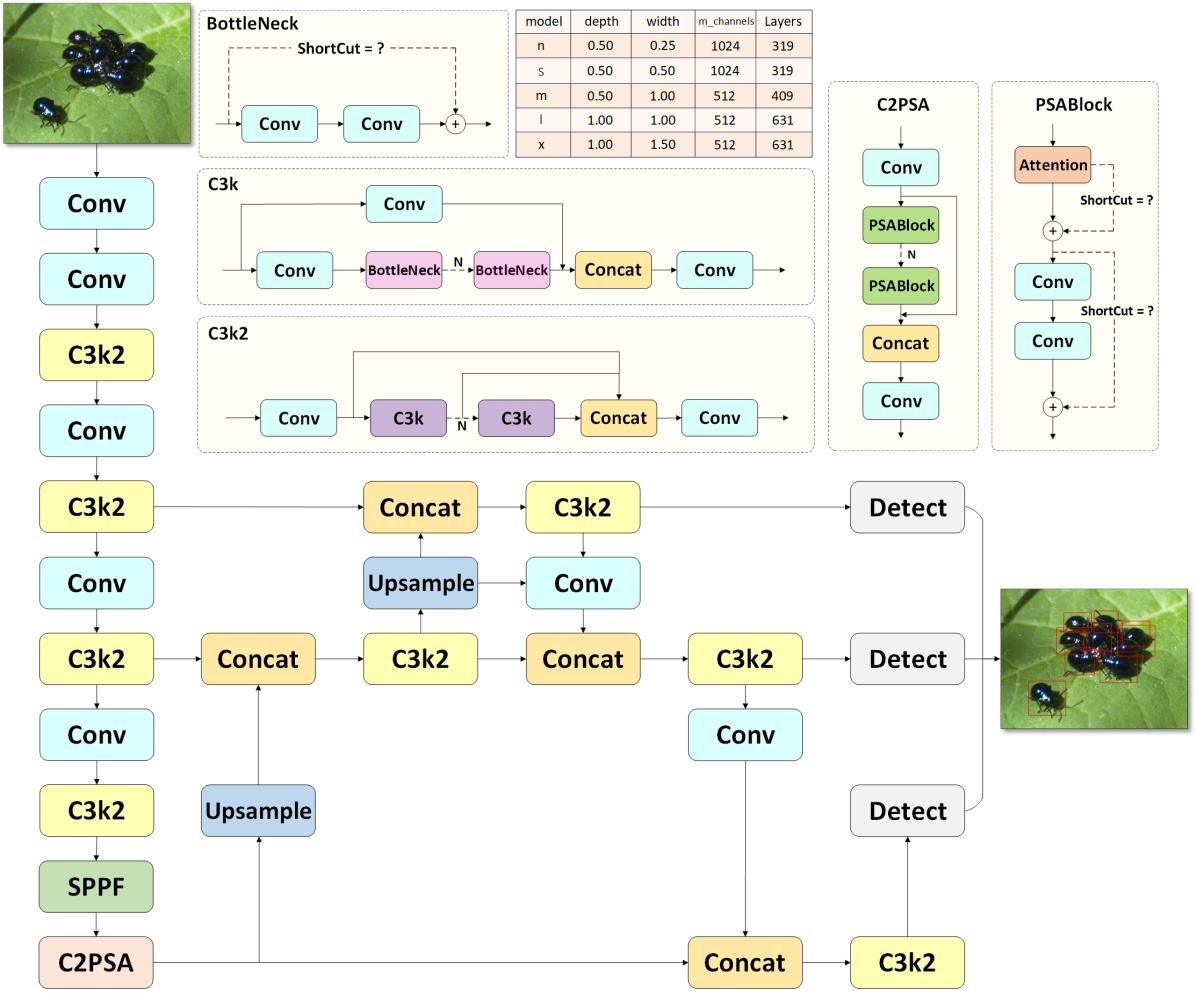
The overall framework of YOLO11 incorporates the BottleNeck, C3k, C3k2, PASBlock, and CSPSA modules, which are responsible for feature extraction within the network. The red box in the figure represents the pest detected. The table presents five variants of the YOLO11 model—n, s, m, l, and x—along with their corresponding network depth, width, maximum number of channels, and number of layers.

In this study, we selected the latest model, YOLO11n [34], as the baseline model. The model offers five variations with different network widths and depths: n, s, m, l, and x. With only 2.6 million parameters and 6.5 GFLOPs in computational load, YOLO11n maintains high accuracy in object detection tasks. As shown in Figure 4, the YOLO11 model architecture consists of five main components: the input module, the backbone network, the neck module, the detection head, and the output module.

The input module of YOLO11 includes several preprocessing steps, such as resizing the input images to a consistent size and performing image transformation and augmentation, as well as other data preprocessing techniques.

The backbone network consists of four modules, including the Conv module, C3k2 module, Spatial Pyramid Pooling-Fast (SPPF) module [35], and C2PSA module, as illustrated in Figure 4. The C3k2 module replaces the C2f module from YOLOv8 with a C3k2 module, which contains C3k blocks. Each C3k block uses a Bottleneck connection to ensure stable gradient flow and progressively enhance feature representation. The SPPF module, as a Spatial Pyramid Pooling module, performs pooling on feature maps at multiple scales without increasing the computational load, thus expanding the model’s receptive field to accommodate objects of various sizes. The C2PSA module is a convolutional block with parallel spatial attention, which computes the similarity between all feature points to enhance feature extraction capabilities.

The neck module of YOLO11 utilizes a feature pyramid network (FPN) and path aggregation network (PAN) structure for the path aggregation of features at different levels. The FPN introduces a top-down feature aggregation pathway that helps retain high-level semantic information, which is typically lost in the deeper layers of networks. Building on the FPN, the PAN adds a bottom-up pathway that enhances the flow of low-level, spatially detailed features toward deeper layers. This improves the localization and detection of small objects by preserving high spatial resolution. The FPN’s top-down and PAN’s bottom-up pathways together enable the neck module to effectively aggregate features across different levels of abstraction.

In the detection head section, YOLO11 separates the localization and classification detection heads, employing depthwise separable convolutions. The output layer, which accounts for variations in object scale, consists of three feature maps at different scales, enabling the detection of objects of varying sizes. Additionally, non-maximum suppression (NMS) is applied to eliminate redundant prediction boxes [36].

### 3.2. SP-YOLO Model

YOLO11 has demonstrated strong performance in general object detection tasks. However, its application to the sugar beet pest detection dataset presents several challenges. These challenges stem primarily from the unique characteristics of agricultural environments, which differ significantly from the typical datasets used to train general-purpose detection models. Specifically, pest species often exhibit significant scale variations, with some pests being very small. Moreover, in complex agricultural environments, pests are often well camouflaged. Some pest species exhibit morphological similarities to the leaves or stems of the plants, further complicating detection. Additionally, variations in lighting, angles, and other environmental factors contribute to the challenges of agricultural pest detection. All these factors increase the likelihood of both false positives and missed detections. Consequently, YOLO11, as a general object detection model, struggles to adapt effectively to these specific challenges in the agricultural context.

**Figure 5:**
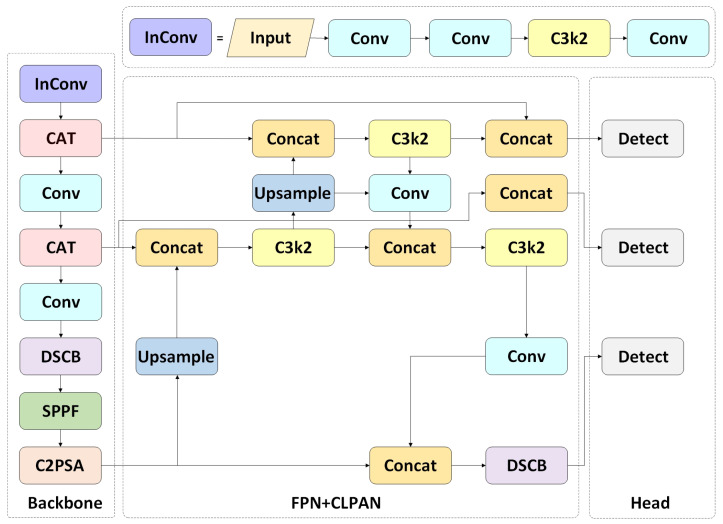
The overall framework of the SP-YOLO model includes a novel backbone network (CAT) for feature extraction, a feature pyramid network (FPN) and a cross-layer path aggregation network (CLPAN) for multi-scale feature fusion, and head components responsible for generating detection bounding boxes at different scales.

To address these limitations, we propose the SP-YOLO model, specifically tailored for sugar beet pest detection. The architecture of the SP-YOLO model is depicted in Figure 5. SP-YOLO optimizes the backbone network by incorporating a transformer module, which enhances the model’s ability to capture long-range dependencies. The use of depthwise separable convolutions further expands the receptive field. Additionally, the integration of low-level features into high-level feature maps improves the model’s performance, resulting in better overall performance compared to YOLO11. The following section will provide a detailed analysis of how these modules address the identified limitations.

#### 3.2.1. Optimization of the Backbone Network

**Figure 6:**
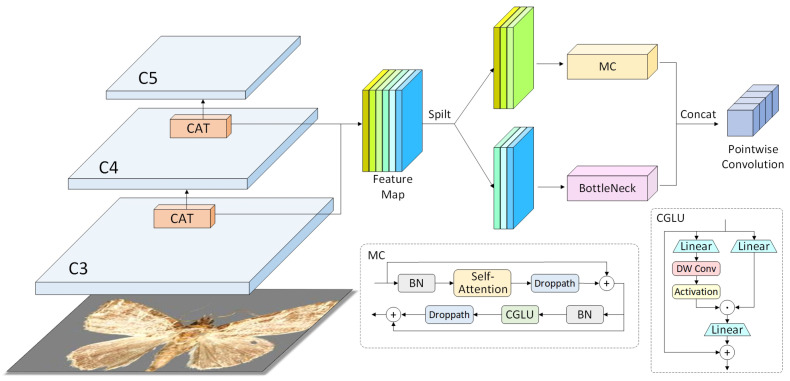
The CAT module is connected after the C3 and C4 layers and incorporates the MC module. The MC module consists of the multi-head self-attention (MHSA) module, the convolutional gated linear unit (CGLU) module, and the BottleNeck module. The input feature maps are processed through the MC module and the BottleNeck module, concatenated, and then undergo feature exchange via pointwise convolution.

The backbone network is pivotal in feature extraction within YOLO11, as its ability to extract effective features directly influences the detection performance of the neck and head modules. YOLO11 employs an efficient and lightweight backbone network, offering strong feature extraction capabilities. However, this backbone struggles to capture long-range dependencies, which are essential for accurate agricultural pest detection. The transformer architecture, known for its robust global feature extraction ability, has been widely adopted for detection tasks. Inspired by the swin transformer [37], this study introduces a hybrid CNN–transformer architecture, referred to as CAT, as shown in Figure 6. In this architecture, the feature map is divided into two parts: one is processed using a standard CNN pipeline to capture local patterns and low-level features, and the other is processed through transformer blocks to model long-range dependencies. The transformer leverages a multi-head self-attention mechanism, which excels at capturing global relationships across different regions of the input feature map. This enables the model to focus on important contextual information from the entire spatial domain.

To mitigate the high computational cost associated with the transformer structure, we introduce a channel selection strategy. Only a subset of the feature channels is processed by the transformer, significantly reducing the computational burden. The remaining channels are processed using convolutional gated linear unit (GLU) modules, which incorporate a gating mechanism to control the flow of information. This mechanism allows each feature or pixel to focus on its local neighbors, thereby enhancing the model’s ability to capture local features. This local attention mechanism is particularly effective in identifying fine-grained details in the feature map, such as edges, textures, and small object details, thereby improving the model’s capacity to highlight relevant features.

The proposed CAT architecture strikes a balance between computational efficiency and feature extraction capability. By combining the strengths of the CNN and transformer, this hybrid approach enables YOLO11 to overcome the limitations of traditional CNN backbones, which struggle to capture global dependencies, while maintaining a lightweight and efficient design.

#### 3.2.2. Addition of Depthwise Separable Convolution Block (DSCB)

**Figure 7:**
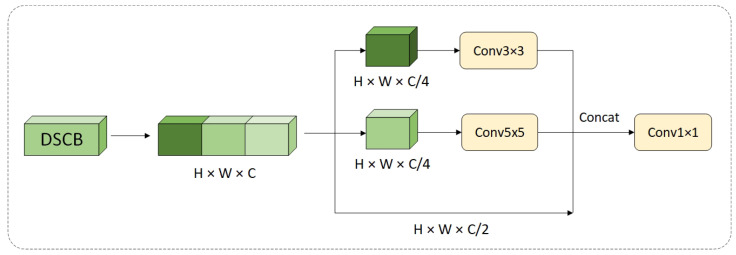
Depthwise separable convolution blocks are organized into three groups. Two of these groups perform convolution operations using 3×3 and 5×5 convolution kernels, respectively, while the third group remains unchanged. Finally, a 1×1 convolution is applied to facilitate channel information exchange.

As the number of layers of the neural network increases, the number of channels increases exponentially, which carry a lot of redundant information. Depthwise separable convolutions (DSCs) are a type of factorized convolution that decomposes a standard convolution into a depthwise convolution followed by a 1 × 1 convolution [38], known as a pointwise convolution. This approach is widely used in devices with limited computational resources. Motivated by the advantages of DSC, the present study introduces a lightweight deep separable convolution module (DSCB), as illustrated in Figure 7.

The input feature maps are divided into three groups. Two of these groups undergo feature extraction using 3×3 and 5×5 convolutional kernels, which are effective in capturing features at different scales. These convolutional kernels also have varying receptive field sizes within the same network layer, enhancing the network’s expressive capacity. The features from the three groups are then concatenated, and a 1×1 convolution is applied to facilitate channel-wise information exchange. This design aims to enable multi-scale feature extraction while reducing the number of parameters.

A comparison of the parameter counts between the standard convolution and the proposed DSCB is provided below.

(1) Calculation of Parameters for Standard Convolution in this Layer: Let the depth of the input image be $C_{\mathrm{in}}$, the size of the convolutional kernel be K×K, and the depth of the output be $C_{\mathrm{out}}$. In this case, the size of the convolutional kernel for each output channel is $C_{\mathrm{in}}\times K\times K$. The total number of parameters is given by
  (1) $$\;\mathrm{Total}\;\mathrm{parameters}\;=C_{\mathrm{in}\;}\times C_{\mathrm{out}\;}\times K^{2}$$
(2) Calculation of Parameters for Depthwise Separable Convolution Block in this Layer:

Assuming the depth of the input image is $C_{\mathrm{in}}$, the size of convolutional kernel 1 is $K_{1}\times K_{1}$, the size of convolutional kernel 2 is $K_{2}\times K_{2}$, and the depth of the output is $C_{\mathrm{out}}$. The number of parameters after applying convolutional kernel 1 and kernel 2 is denoted by $S_{1}$ and $S_{2}$. The number of parameters for the pointwise convolution is denoted by $S_{3}$.

(2) $$S_{1}=C_{\mathrm{in}\;}\times C_{\mathrm{out}\;}\times K_{1}^{2}\times 0.25$$

(3) $$S_{2}=C_{\mathrm{in}\;}\times C_{\mathrm{out}\;}\times K_{2}^{2}\times 0.25$$

(4) $$S_{3}=1\times 1\times \left(0.5\times C_{\mathrm{in}\;}+2\times C_{\mathrm{out}\;}\right)\times C_{\mathrm{out}\;}$$

The total number of parameters for the DSCB is $S_{1}+S_{2}+S_{3}$.

(5) $$\;\mathrm{Total}\;\mathrm{parameters}\;=C_{\mathrm{in}\;}\times C_{\mathrm{out}\;}\times 0.25\times \left(K_{1}^{2}+K_{2}^{2}\right)+\left(0.5\times C_{\mathrm{in}\;}+2\times C_{\mathrm{out}\;}\right)\times C_{\mathrm{out}\;}$$

The ablation experimental results show that replacing the conventional convolution of feature map P5 with the DSCB reduced the number of parameters in the convolution operation of this layer by 13.8%, while the mean average precision (mAP@50) increased by 2.4%.

#### 3.2.3. Cross-Layer Path Aggregation Network (CLPAN)

In YOLO11n, the neck module employs the FPN [39] and PAN [40] structures. The FPN is a top-down architecture that enhances high-level features by upsampling and combining them with low-level features to generate prediction maps. The PAN extends the FPN by adding a bottom-up feature pyramid, aggregating features from different backbone layers into various detection layers. This dual-pyramid structure improves both semantic understanding and localization accuracy. However, as information passes through the FPN and PAN structures, finer details, such as contours, textures, and colors associated with pest infestations, are gradually lost as the network deepens. This loss of information hinders the effective enhancement of multi-scale features, ultimately reducing the network’s ability to detect sugar beet pests across different scales.

**Figure 8:**
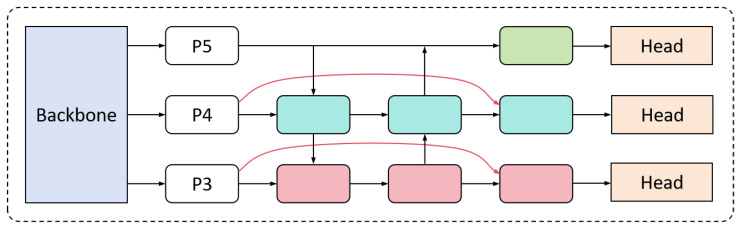
The structure of the CLPAN module: red arrows in the figure represent cross-layer feature fusion. The P3 and P4 feature maps from the backbone are connected to higher-level features via cross-layer connections, enabling multi-scale feature fusion through cross-layer information exchange.

To address this limitation, the proposed cross-layer path aggregation network (CLPAN) extends the FPN framework with additional enhancements, as illustrated in Figure 8. In this study, the feature layers Conv3, Conv4, and Conv5 are selected as inputs for the FPN. The low-level features extracted by the backbone network are represented as P3, P4, and P5, with the strides of these feature layers relative to the original feature map being 8, 16, and 32, respectively. Then, the features extracted from P3 and P4 are fused with those represented by the original PAN. Three scales of feature maps are output to detect small-scale beet pests by small feature maps and large-scale beet pests by large-scale feature maps. The proposed cross-layer feature aggregation mechanism enables the network to effectively capture contextual information across multiple scales, enhancing its receptive field at different spatial resolutions. This approach preserves the low-level features crucial for detecting fine-grained details while integrating higher-level semantic context. Specifically, when object scales vary significantly, the fusion of low-level features with rich high-level context improves the model’s ability to differentiate between small and large objects. Ablation experiments demonstrate that the CLPAN structure improves the model’s mAP@50 by 2.5%.

## 4. Experimental Results and Analysis

### 4.1. Experimental Environment and Parameter Setting

All experiments in this study were conducted on an Ubuntu 22.04 operating system, equipped with an Intel(R) Xeon(R) Platinum 8362 CPU @ 2.80 GHz, 45G RAM, and two RTX 3090 GPUs, each with 24 GB of graphics memory. The models were developed using PyTorch 2.1.2 and Python 3.8 with CUDA 11.8 and CUDNN 8.6.0. The experimental environment configuration is detailed in Table 3.

Before model training, it is essential to appropriately configure the hyperparameters, as they can have a significant impact on model performance. As shown in Table 4, lr0 and lrf represent the initial and final learning rates, respectively. A typical starting value for the initial learning rate is 0.01, although adjustments may be required based on training dynamics. If the model experiences slow convergence or oscillations in the early stages, reducing the learning rate can help. Conversely, if the model converges too early to a suboptimal local minimum, increasing the learning rate may facilitate better exploration of the parameter space. The Stochastic Gradient Descent (SGD) optimizer is employed to adjust the learning rate during training, with the momentum set to 0.937, a batch size of 32, and an input image resolution of 640 × 640 pixels. The Intersection over Union (IoU) threshold is set to 0.7 to evaluate the overlap between predicted and ground truth bounding boxes. The model is trained for 300 epochs, with an early stopping criterion implemented: if no improvement in accuracy is observed for 100 consecutive epochs, training will be terminated.

### 4.2. Evaluation Metrics

This study evaluates the model’s accuracy in detecting sugar beet pests using mAP@50, mAP@50:95, and average precision (AP). mAP@0.5 represents the mean average precision (mAP) at an IoU threshold of 0.5, while mAP@50:95 refers to the average mAP across IoU thresholds from 0.5 to 0.95, with a step size of 0.05, resulting in a total of ten steps. The final value is the sum of these steps’ averages. Recall (R) is used to assess the completeness of the predictions. The model’s complexity is evaluated based on the number of parameters (Params[M]) and the computational cost (GFLOPs). The model’s inference speed is measured using PFS. The detailed calculation formulas are provided below.

(6) $$P=\frac{TP}{TP+FP}$$

(7) $$R=\frac{TP}{TP+FN}$$

(8) $$AP=\int_{0}^{1}P\left(R\right)dR$$

(9) $$mAP@50=\frac{\sum_{i=1}^{b}AP_{i}}{b}$$

(10) $$mAP@50:95=\frac{mAP@50+mAP@55+\cdots +mAP@95}{10}$$

### 4.3. Comparison Experiments of Different Object Detection Models

#### 4.3.1. Comparison with Advanced Models

To provide a more intuitive understanding of the detector’s performance, this paper presents the results of SP-YOLO in sugar beet pest detection and compares it with several state-of-the-art algorithms, including Faster R-CNN [12], RetinaNet [16], FCOS [17], DETR [41], RTMDet [42], YOLOv5n [43], YOLOXn [44], YOLOv8n [45], YOLOv10n [46], and YOLO11 [34], as shown in Table 5.

The proposed SP-YOLO demonstrates excellent performance in sugar beet pest detection. Specifically, SP-YOLO achieves a mAP@50 of 0.884, a mAP@50:95 of 0.612, a precision (P) of 0.887, and a recall (R) of 0.831. Among these metrics, SP-YOLO exhibits the best overall performance. The model has a computational cost of 2.8 GFLOPs and 8.5 million parameters. Compared to YOLO11n, SP-YOLO increases the parameter count by 7.7%, yet it improves mAP@50 by 4.9%, AP by 9.9%, and recall by 1.3%. Furthermore, the overall parameters and computational cost of SP-YOLO are well-controlled, remaining significantly lower than those of models such as Faster R-CNN, SSD, RetinaNet, and FCOS. This balance between performance and efficiency is thus highly advantageous. Additionally, SP-YOLO achieves a frame rate of 136 FPS, making it suitable for real-time detection tasks.

**Figure 9:**
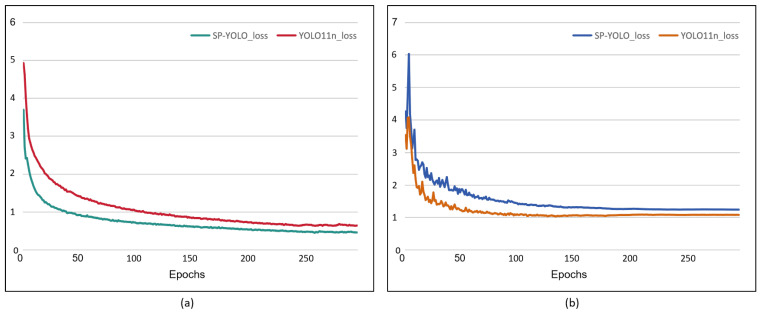
Comparison of the classification loss between YOLO11n and SP-YOLO. (**a**) Train classification loss; (**b**) val classification loss.

**Figure 10:**
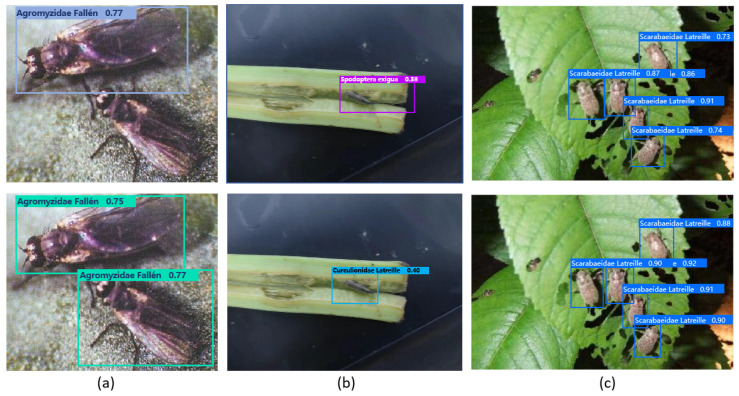
The visualization comparison of detection performance between YOLO11n (**Top**) and SP-YOLO (**Bottom**) shows that YOLO11n exhibits a higher number of (**a**) missed detections and (**b**) false positives. In contrast, SP-YOLO demonstrates higher confidence in (**c**) dense pest images.

Figure 9 presents the training and validation loss curves for SP-YOLO in comparison with the baseline YOLO11n. The results indicate that SP-YOLO achieves lower loss values and converges more rapidly than YOLO11n. Additionally, Figure 10 compares the detection results of YOLO11n and SP-YOLO on the BeetPest dataset. YOLO11n exhibits more missed detections and false positives compared to SP-YOLO. In images with dense pest populations and occlusions, SP-YOLO demonstrates higher confidence in its predictions.

#### 4.3.2. Replacement of the Backbone Network

This study evaluates several advanced backbone networks, including StarNet [47], MobileNetV4 [48], ConvextV2 [49], FasterNet [50], and EfficientVit [51], as summarized in Table 6. Compared with the proposed YOLO11n-CAT, the results show that YOLO11n-CAT achieves the best performance in mAP@50 and mAP@50:95. It ranks second in precision (P), following FasterNet, a lightweight network introduced in 2023, which efficiently extracts spatial features by minimizing redundant computations and memory access. In terms of recall (R), YOLO11n-CAT is slightly outperformed by EfficientVit, a vision transformer-based network that achieves a balanced trade-off between accuracy and computational speed. The parameters and GFLOPs of YOLO11n-CAT are second only to StarNet, a lightweight backbone proposed by Microsoft in 2024, but while StarNet has the fewest parameters and computations in this study, it performs poorly in terms of recognition accuracy. The use of the CAT-enhanced backbone network demonstrates advantages in global feature extraction, enabling it to capture network long-range dependencies. Based on its overall performance, the CAT-improved YOLO11n is selected as the optimal backbone network for this task.

### 4.4. Ablation Experiments

**Figure 11:**
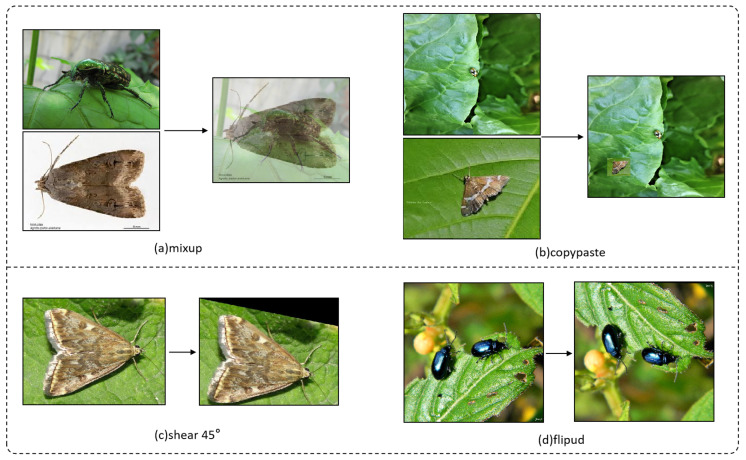
Four data augmentation techniques.

Data augmentation (DA) is an effective method for enhancing model accuracy without increasing the number of parameters or computational complexity. However, it does come with the drawback of extended training times. To assess the impact of the proposed module, four data augmentation strategies are employed in this study: mixup, copy–paste, shear 45°, and vertical flipping (flipud), each applied with a probability of 0.5. As illustrated in Figure 11. It is noteworthy that the YOLO11n model already incorporates Mosaic 1.0 data augmentation, random erasing with a probability of 0.4, and horizontal flipping (fliplr) with a probability of 0.5.

To validate the effectiveness of DA, CAT, DSCB, and CLPAN, this study conducted ablation experiments. The experimental results are presented in Table 7, which details the application of each block. Four data augmentation techniques were applied solely to the training set. Compared to YOLO11n, mAP@50 improved by 2.4%, mAP@50:95 increased by 3.1%, and both P and R showed improvements of 1.7% and 2.0%, respectively. After incorporating the CAT, DSCB, and CLPAN, mAP@50 increased by 2.8%, 2.4%, and 2.5%, respectively, compared to YOLO11n. precision (P) increased by 1.1%, 2.0%, and 1.0%, while recall (R) saw gains of 3.2%, 0.4%, and 3.3%, respectively. The backbone network constructed with the CAT and DSCB exhibits high accuracy and reliability, and the CLPAN effectively captures multi-scale variations in pest detection. The significant improvements in recall suggest that the model is better able to detect true targets, thereby minimizing missed detections. Notably, the positive impact of the DSCB on the model’s performance is contingent upon a sufficiently high number of channels, with excellent performance observed when the number of channels exceeds 1024.

Furthermore, ablation experiments using pairwise combinations, as well as all three modules together, consistently delivered strong results across all evaluation metrics. Ultimately, SP-YOLO demonstrated the best performance, outperforming other models in terms of mAP@50, precision, and recall.

### 4.5. Validation of Model Generality

The RTB dataset consists of images of six common invasive pine tree pests—boerner, armandi, coleoptera, linnaeus, leconte, and acuminatus—captured under controlled laboratory conditions in Petri dishes, with a total of 2183 images. The pest species in the dataset exhibit a high degree of similarity, posing a significant challenge for accurate detection. To assess the robustness and generalizability of the proposed model, experiments were conducted on the RTB dataset. The detection performance of SP-YOLO and the YOLO series is summarized in Table 8. Compared to other models, SP-YOLO achieves superior performance in terms of mAP@50 and mAP@50:95. It exhibits exceptional performance on datasets characterized by pest specimens with similar morphological features, highlighting its robustness and versatility as an effective model for pest detection.

## 5. Conclusions

This paper introduces a high-precision and efficient multi-scale SP-YOLO model for the real-time detection of agricultural pests in complex field environments. The backbone of PB-YOLO improves the YOLO11n backbone network by incorporating the CAT module, which combines convolutional neural networks with a transformer. This fusion not only strengthens the feature extraction capabilities of the network but also improves its ability to capture long-range dependencies. The DSCB module is embedded into the network, enhancing its receptive field at different spatial resolutions and improving detection accuracy. In the neck section, considering the loss of low-level features after multiple convolutional layers, innovative modifications are applied to the FPN and PAN structures, leading to the development of the CLPAN. This multi-level feature aggregation mechanism enables the network to capture contextual information at different scales while preserving and integrating low-level features, thereby enhancing the multi-scale target detection accuracy of the model. Although the inclusion of the CLPAN module results in a slight increase in both the parameter count and computational cost compared to YOLO11n, SP-YOLO outperforms other models across various detection metrics, thereby justifying this trade-off.

The BeetPest dataset, as proposed in this study, exhibits a class imbalance, with 770 images in the Agrotis category compared to only 114 images in the "traces of damage" category. This disparity in sample size may lead to biased model training. To alleviate this issue, data augmentation techniques were employed during the SP-YOLO training process. However, addressing class imbalance remains a challenge, and future research should focus on the development of more robust and effective strategies to mitigate this limitation.

Future research should focus on the following directions:

(1) Pest Damage Traces: Traces of pest damage provide valuable and trackable information. In this study, we successfully identified the damage caused by beet leaf miners on beet leaves, yielding promising recognition results. Expanding this approach to capture pest damage traces across multiple pest species will aid farmers in pinpointing pest locations and applying more targeted interventions.
(2) Multimodal Data Integration: Integrating both image and textual data can further enhance the model’s performance. Relying solely on image modality often proves insufficient for accurate pest detection, as it does not fully capture the complex variations in pest characteristics. By incorporating textual descriptions of pests, a multimodal model can be developed, which has more practical significance for pest detection in real-world agricultural environments.

## Figures and Tables

**Table 1:** Three key challenges with existing pest detection models.

Papers	Deficiencies of the Model	Cause
[22,23,24,25,26]	A large number of parameters, high computational effort, slow model inference.	Models require significant memory and storage resources, which hinders their deployment on edge devices and limits their ability to support real-time detection. These factors restrict their applicability in certain scenarios.
[19,27,28,29]	Trained on a limited number of pest species and a single pest scale.	Pest images exhibit considerable diversity in species and scale, which can impair the model’s ability to generalize across different pest types and adapt effectively to varying scales.
[22,29,30,31]	The dataset image background is too simple.	Models struggle with adapting to environmental changes in complex field conditions, leading to suboptimal performance.

**Table 2:** The thirteen categories, abbreviations, and number of images in the BeetPest dataset.

Name	Internet Images	IP102	Field Photography	Total
Scarabaeidae Latreille, 1802	128	203	99	430
Curculionidae Latreille, 1802	149	383	149	681
Chrysomelidae Latreille, 1802	334	345	23	702
Agromyzidae Fallén, 1823	137	59	4	200
*Loxostege sticticalis* Linnaeus, 1761	117	424	0	541
*Spoladea recurvalis* (Fabricius, 1775)	458	0	0	458
*Spodoptera litura* (Fabricius, 1775)	238	413	0	651
*Mamestra brassicae* (Linnaeus, 1758)	204	0	10	214
*Agrotis* Ochsenheimer, 1816	317	453	0	770
*Spodoptera exigua* (Hübner, 1808)	109	0	86	195
*Helicoverpa armigera* (Hübner, 1808)	82	0	45	127
*Discestra trifolii* (Hufnagel, 1766)	0	0	235	235
Traces of damage	0	0	114	114
Total	2273	2280	765	5318

**Table 3:** Experimental environment configuration.

Configuration	Parameter
Operating system	Ubuntu 22.04
Cpu	Intel(R) Xeon(R) Platinum 8362 CPU @ 2.80 GHz
Gpu	Two RTX 3090 (24 GB)
RAM	45 GB
Languages	Python 3.8
Framework	PyTorch 2.1.2
Operating platform	CUDA 11.8 and CUDNN 8.6.0

**Table 4:** Hyperparameters for model training.

Name	Value	Name	Value
lr0	0.01	Batch	32
lrf	0.01	imgsz	640
Optimizer	SGD	IoU	0.7
Momentum	0.937	epochs	300

**Table 5:** Comparison of SP-YOLO with some generic object detection algorithms on BeetPest. Bold values indicate optimal results.

Method	mAP@50	mAP@50:95	P	R	GFLOPs	Param(M)	FPS
Faster R-CNN [12]	0.845	0.561	0.711	0.664	178	41.4	30
SSD-300 [15]	0.874	0.588	0.832	0.656	31	25.3	99
RetinaNet [16]	0.833	0.582	0.619	0.718	178	36.5	34
FCOS [17]	0.789	0.518	0.805	0.698	168	32.1	33
Detr [41]	0.812	0.585	0.827	0.8	82	41.5	32
RTMdet [42]	0.847	0.603	0.732	0.65	8	4.8	36
YOLOv5n [43]	0.833	0.577	0.821	0.816	4.2	1.77	103
YOLOXn [44]	0.746	0.405	0.759	0.506	**0.524**	**0.899**	69
YOLOv8n [45]	0.842	0.592	0.842	0.758	8.1	3	82
YOLOv10n [46]	0.826	0.579	0.816	0.762	8.3	2.7	123
YOLO11n [34]	0.835	0.596	0.788	0.818	6.3	2.6	**154**
SP-YOLO (Ours)	**0.884**	**0.612**	**0.887**	**0.831**	8.5	2.8	136

**Table 6:** Experimental comparison results between CAT backbone network and recent advanced backbone networks. Bold values indicate optimal results.

Backbone	mAP@50	mAP@50:95	P	R	GFLOPs	Param(M)
Starnet [47]	0.789	0.543	0.794	0.729	**1.9**	**5**
Mobilenetv4 [48]	0.798	0.563	0.737	0.746	5.4	21
Convextv2 [49]	0.812	0.561	0.759	0.758	5.4	12.6
Fasternet [50]	0.827	0.572	**0.845**	0.734	3.9	9.2
EfficientViT [51]	0.856	0.598	0.804	**0.829**	3.7	7.9
YOLO11n [34]	0.835	0.596	0.818	0.788	2.6	6.3
YOLO11n-CAT	**0.863**	**0.626**	0.829	0.82	2.5	6.3

**Table 7:** Results of ablation experiments. “✓” denotes module activation, while “×” denotes module deactivation. Bold values indicate optimal results.

DA	CAT	DSCB	CLPAN	mAP@50	mAP@50:95	P	R
×	×	×	×	0.835	0.596	0.818	0.788
✓	×	×	×	0.859	0.627	0.835	0.808
✓	✓	×	×	0.863	0.626	0.829	0.82
✓	×	✓	×	0.859	0.622	0.838	0.792
✓	×	×	✓	0.860	0.621	0.828	0.821
✓	✓	×	✓	0.864	0.607	0.84	0.818
✓	✓	✓	×	0.869	0.624	0.824	0.822
×	✓	✓	✓	0.871	**0.632**	0.834	0.809
✓	✓	✓	✓	**0.884**	0.612	**0.887**	**0.831**

**Table 8:** Comparison of YOLO11n and SP-YOLO on the RTB dataset. Bold values indicate optimal results.

Model	mAP@50	mAP@50:95
YOLOv5n	0.861	0.618
YOLOXn	0.763	0.536
YOLOv8n	0.851	0.615
YOLOv10n	0.855	0.622
YOLO11n	0.812	0.611
SP-YOLO(ours)	**0.869**	**0.633**

## Data Availability

The pest images are available at https://github.com/joecreator/Pest (accessed on 3 December 2024). The IP102 dataset is available at [33]. The RTB dataset is available at [32].
